# Study on roof movement law of local filling mining under peak cluster landform

**DOI:** 10.1038/s41598-023-41505-7

**Published:** 2023-09-07

**Authors:** Hengyu Su, Chang Luo, Yichao Jia, Ziyi Wang

**Affiliations:** 1 Guizhou Minzu University, Guiyang, 550025 Guizhou China; 2 Guizhou University, Guiyang, 550025 Guizhou China; 3 Guizhou Heze Engineering Management Consulting Co., Ltd., XingYi, 562400 Guizhou China; 4 Key Laboratory of In-Situ Property Improving Mining of Ministry of Education, Taiyuan University of Technology, Taiyuan, 030024 China

**Keywords:** Civil engineering, Environmental sciences, Engineering

## Abstract

The strip structure filling mining technology is suggested in response to the environmental issues such as surface subsidence and landslides brought on by the mining of 11,071 working faces in a mine in Guizhou. The mining technology system is studied through indoor testing, numerical simulation, and engineering monitoring. According to theoretical study, the filling strip can be steadily loaded and its value doesn't exceed 10 m when the width of the filling strip and the width of the filling interval are set to be equal. According to laboratory testing, fly ash can replace some of the cement in the cement mixture as a binder to maintain strength while cutting costs. The degree of crystallization gradually distributed into the network in the filling paste of various ages corresponds to its strength when combined with the findings of scanning electron microscopy; The numerical simulation results show that the maximum subsidence of the immediate roof is reduced from 340 to 3 mm from the filling rate of 0 to 100%, the filling effect is remarkable, and the shape of the settlement curve is changed from 'U' to 'basin', then to 'W'; during the local filling mining, the settlement curve of the immediate roof presents a 'wave' shape, and the stress curve of the immediate roof in the middle of the stope is also changed. The peak tension of the coal wall falls synchronously with filling spacing on both sides of the stope. The overall vertical stress below the mountain is larger, and the vertical stress at the top of the filling body eventually shifts from a "saddle" shape to a "inverted U" shape without zero support stress. In conjunction with the plastic zone, it is discovered that the stable bearing of the "filling strip-direct roof" composite structure increases with decreasing tensile and shear damage range of the hollow roof area and both sides of the top of the "filling 3 m interval 3 m" scheme; engineering measurement also reveals that the higher the position of the survey line is, the smaller the displacement is. However, the overall displacement of the strata directly above is negligible, and the greatest displacement is only 10.9 mm, which is consistent with the numerical simulation. At the same time, the displacement beneath the mountain area is too great.

## Introduction

The steady depletion of coal resources in eastern China has created an opportunity for long-term development in the western coal energy sector. The low and middle mountain peak cluster landform, which serves as a representative area of Guizhou Province, is home to the majority of the coal mines. The region's geological structure is incredibly complicated, with numerous levels including coal seam occurrences. The long-term, extensive management style of coal mining companies has led to an increase in the prevalence of a number of environmental issues, including coal gangue contamination, mountain collapse, and surface subsidence. Roads for green mining are essential^1,2^.

Many academics have undertaken a number of research on partial fill mining in recent years. The continuous mining and filling paste filling technology was proposed by Skrzypkowski et al.^3–5^. They described the technology's principles and steps, performed a numerical simulation to examine the surface movement characteristics of localized filling mining under the hard top plate of shallowly buried coal seams, and then arrived at the superposition calculation method of filling strip mining based on the probability integral method and provided the formula for surface movement prediction. Similar material simulation experiments and theoretical analysis techniques were used by Yang et al.^6^ to investigate the mechanical mechanisms underlying the spatial and temporal evolution of the stress field, displacement field, and fissure field of the roof plate. They also proposed filling and roof control countermeasures for the in-situ mining airspace area and the middle and high rock layer airspace area. Jia and He^7,8^ adopted the theory of elastic foundation beams, established the displacement equation of the roof plate, and used the mechanical equivalent conversion to derive the mathematical relationship equations between the maximum spanning distance of the roof plate, the distance between the roof control and the filling spacing, a roof control distance, and a filling spacing distance. With the engineering background of partial filling mining in coal mines, Luo et al.^9–11^ investigated the strength of appropriate filling bodies using theoretical calculation and numerical simulation.

Although many scholars have conducted relevant studies on localized infill mining, the mining disturbance under the peaked terrain is more complex than in the plains, and there are certain differences in the movement patterns of the rock layers overlying the mining field^13,14^, so there are fewer studies on localized infill mining under the peaked terrain. As a result, using 11,071 working face of a mine in Liupanshui, Guizhou Province as the engineering background, this paper analyzes the stability of roof transport and surrounding rock of local filling mining under the Peak Tussock geomorphology using indoor tests, numerical simulation, and on-site measurement, among other methods. It reveals the changing rules of strength evolution and microstructure of "coal-based solid waste" filling materials through indoor testing; it analyzes the moving law of the overlying rock layer and its stress characteristics of different filling schemes using numerical simulation software, provides the optimal filling scheme, and performs on-site testing for validation.

## Project profile

**Figure 1:**
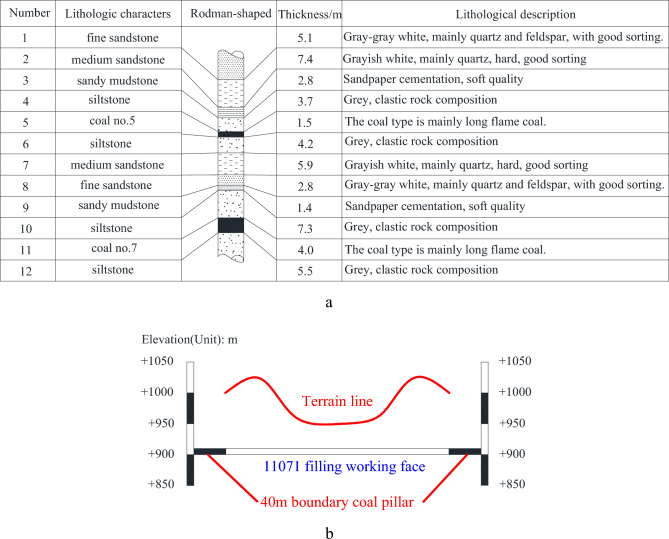
Stratigraphic map and surface elevation profile map. (**a**) The stratigraphy map. (**b**) Surface strike profile in the middle of the stope.

The research area is situated in the Guizhou Province's Liupanshui mining region. It is a landform in a mid-mountain region created by tectonic erosion. The internal terrain has a wide range of elevations, from + 890 m to + 1755.73 m. The mountains are steep, and there are bimodal clusters on the surface. With a strike length of roughly 300 m and a working face length of 100 m, the present mining face is 11,071 working face. The coal seam has an average thickness of about 3 m, a lowest buried depth of about 75 m, and an upper distance from coal seam no. 5 of about 20 m. Mudstone gangue can be mined in 0–3 layers throughout the entire area. The direct floor is mostly silty mudstone, and the direct ceiling is mostly argillaceous siltstone and siltstone. It is anticipated that mining of the 11,071 mining face will result in the sinking and destruction of the provincial highway and the collapse of the mountain, which will cause geological disasters. This is because there are two basic symmetrical barren hills with steep slopes and S212 provincial highways in the upper part of the surface. Later, the mine made the decision to recover shallow resources by using infill mining. Figure 1 depicts a portion of the strata of the coal and rock complex.

## Filling process and theory

### Filling process

**Figure 2:**
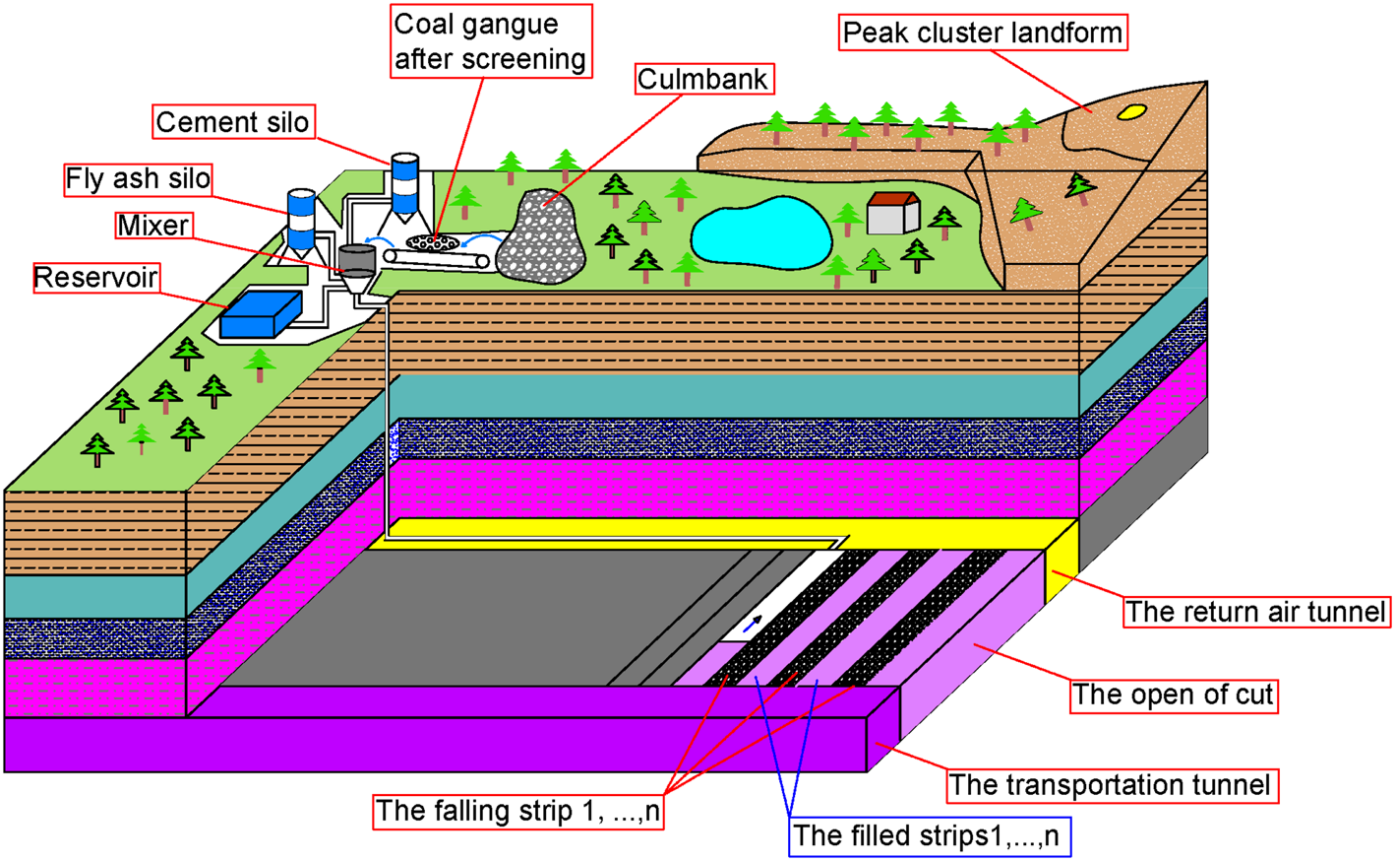
Filling process.

In the working face, the author opts for a small strip filling mining technique, or small strip structure filling mining technique^14,15^. This technique enables underground filling with mining and is a type of underground filling mining technology with high stability and low filling rate. The movement of the strata above is effectively controlled by laying a strip filling body at the critical goaf location, as shown in Fig. 2.

The mining process of small strip structure filling mining method is to arrange the working face according to the wall mining production system, first by making the working face transportation chute, return air chute, and cutting eye through, forming a full negative pressure ventilation system; divide the working face into a number of strips according to the sequence, and mine according to the sequence of "filling strips, 1-collapsed strips, 1-collapsed strips, 2-collapsed strips, 2-collapsed strips, 2-collapsed strips, 2-collapse. The process use coal gangue and fly ash as the primary filling materials, which are blended with cementitious materials and water, mixed in a specific proportion to make a high-concentration slurry, and then sent underground by pipeline. It has cheap mining costs, low system investment, a modest roof area exposed at one time, and an excellent surface subsidence control effect. It is appropriate for controlling surface subsidence and treating solid wastes such as gangue in coal resource mining in western sensitive environment areas.

### Theoretical analysis of filling and caving strip width

**Figure 3:**
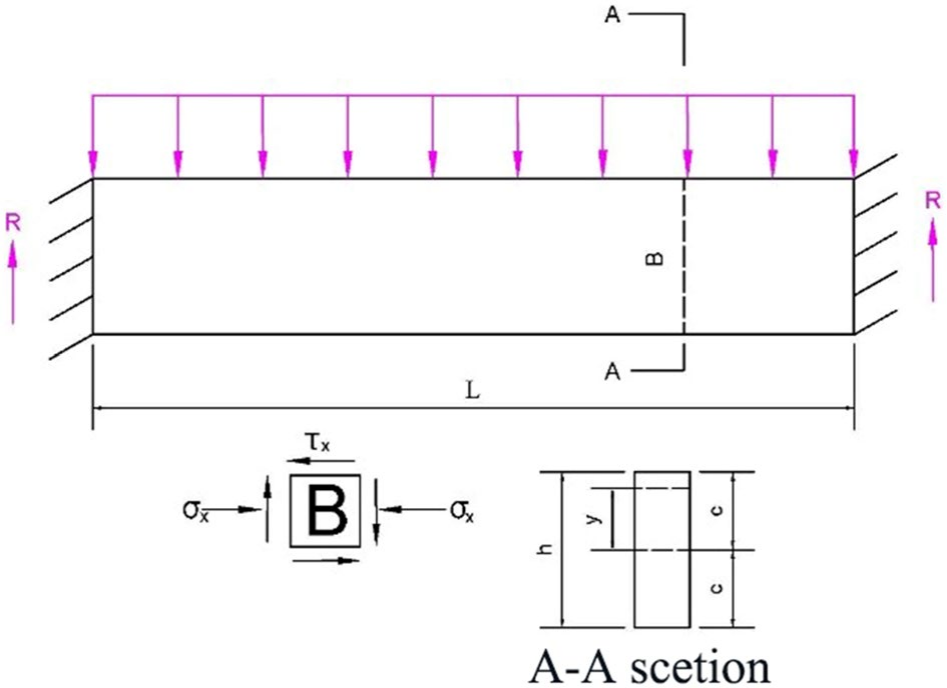
Mechanical model of strip roof fixed beam.

The strip roof rock beam is approximately in a fixed beam state when partially filled. The mechanical model is displayed in Fig. 3^9^ when the roof rock beam is reduced to a fixed beam.

For a rock beam with unit width, the normal stress *σ* and shear stress $\tau xy$ at any point B in the beam are:

1 $$\sigma = \frac{12My}{{h^{3} }}$$

2 $$\tau_{xy} = \frac{3}{2}Q_{x} \left( {\frac{{h^{2} - 4y^{2} }}{{h^{3} }}} \right)$$

3 $$M_{\max } = - \frac{1}{12}ql^{2}$$

4 $$Q_{\max } = \frac{ql}{2}$$

In formula (2), *M* and Q _*x*_ are the bending moment and shear force of the section where point B is located, *y* is the distance from point B to the neutral axis of the section, *h* is the thickness of the beam, *q* is the load of overlying strata, and *l* is the length of the filling zone. The maximum bending moment and shear stress $Q\max$ occur at both ends of the beam.

The maximum tensile stress $\sigma \max$ and the maximum shear stress $(\tau xy)\max$ are:

5 $$\sigma_{\max } = \frac{{ql^{2} }}{{2h^{2} }}$$

6 $$\tau_{\max } = \frac{3ql}{{4h}}$$

When the maximum tensile stress $\sigma \max$ reaches the ultimate tensile strength $RT$ of the main roof or the maximum shear stress $(\tau xy)\max$ reaches the ultimate shear strength $RS$ of the main roof, the main roof will crack. At this point, the limit span $a1T$ and $a2S$ under the constraint of the maximum tensile stress and the maximum shear stress are respectively:

7 $$a_{1T} = h\sqrt {\frac{{2R_{T} }}{q}}$$

8 $$a_{2S} = \frac{{4hR_{S} }}{3q}$$

The roof stratum is regarded as a fixed plate around. When the roof is in the ultimate suspended state, the maximum principal bending moment $M\max$ occurs in the middle of the long fixed edge.

9 $$\left| {M_{\max } } \right| = \frac{{qa^{2} \left( {1 - u^{2} } \right)^{2} \left( {1 + u\lambda^{2} } \right)}}{{12\left( {1 + \lambda^{4} } \right)}}$$

10 $$M_{\max } = \frac{{h^{2} R_{T} }}{6}$$

where *u* is the Poisson 's ratio of the basic vertex; *q* is the basic top weight and its overlying load; $\lambda \, = \, a \, / \, l$ is the geometric shape parameter of the goaf, and *a* is the width of the mining strip.

The formula (9) and (10) are combined to obtain the calculation formula of the limit span $a3T$ of the basic roof fracture under the boundary condition of the surrounding fixed support:

11 $$a_{{3{\text{T}}}} = \frac{h}{{1 - u^{2} }}\sqrt {\frac{{2R_{T} \left( {1 + \lambda^{4} } \right)}}{{q\left( {1 + u\lambda^{2} } \right)}}}$$

For sufficient safety, the minimum value here is taken as the limit width a of the mining strip.

12 $$a = \min \left\{ {a_{1T} ,a_{2S} ,a_{3T} } \right\}$$

According to the field investigation, *l* is 10 m, the average bulk density of overlying strata *γ* is 25 kN/m^3^, the thickness of the main roof is 5.0 m, and the Poisson 's ratio u is 0.25, which are brought into formula (3)–(11), and $a1T$, $a2S$ and $a3T$ are 10, 10 and 13.5 respectively. The limit width of the mining strip is 10 m.

**Figure 4:**
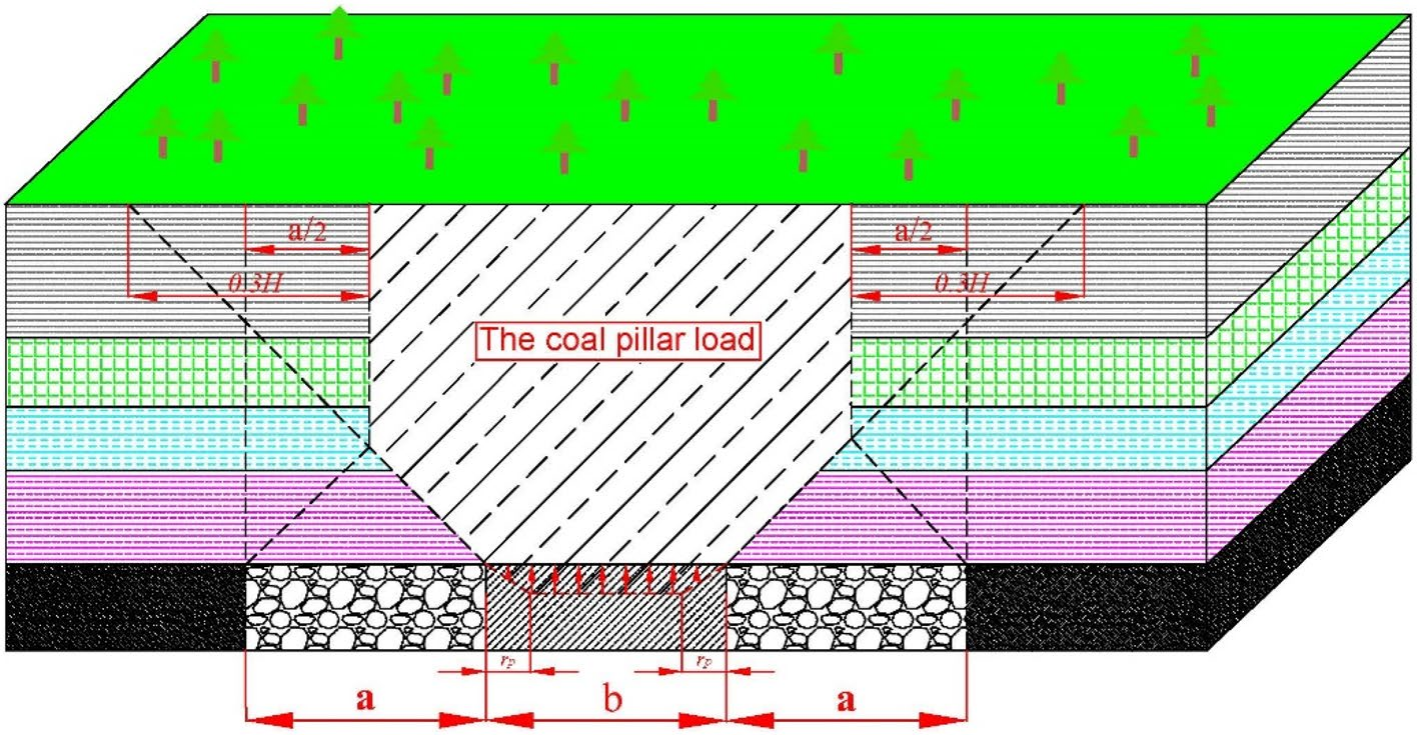
Load distribution when the width of goaf on both sides of coal pillar/ filling zone is less than 0.6H.

By Wilson theory^16^, when the width of the goaf on both sides of the strip coal pillar/filling zone is less than 0.6H, as shown in Fig. 4, the load *Fs* of the strip coal pillar/filling zone per unit length is:

13 $$F_{s} = \gamma H\left[ {b + a\left( {1 - \frac{5a}{{6H}}} \right)} \right]$$

In the formula: γ is the average bulk density of the overlying strata, H is the mining depth, b is the width of the coal pillar/filling zone. Under the gravity pressure of the upper composite rock beam, some areas on both sides of the coal pillar/filling zone are in plastic deformation state, and the middle area is in elastic state, which is often called ' elastic core ' coal pillar. The vertical stress inside the strip coal pillar/filling belt is inverted ' saddle shape ', research shows that^17^, the vertical stress can be approximately replaced by isosceles trapezoid load, as shown in the red trace in Fig. 4.

When there is a plastic zone, the maximum load Fp that the coal pillar can bear is:

14 $$F_{p} = \frac{1}{2}\left[ {\left( {b - 2r_{p} } \right) + b} \right]\sigma_{p}$$

In the formula (14), $\sigma p$ is the strength of coal pillar/filling pillar, $rp$ is the width of plastic zone of filling strip. The solution equation of $rp$ is:

15 $$r_{p} = \frac{MAd}{{2\tan \varphi_{0} }}\ln \left( {1 + \frac{K\gamma H\cos \alpha }{{c_{0} }}\tan \varphi_{0} } \right)$$

In the formula: *M* is the height of coal pillar/filling column, *A* is the lateral pressure coefficient, *d* is the mining disturbance coefficient, generally 1.5 − 3.0, *K* is the maximum stress concentration coefficient on the coal pillar/filling column, *α* is the dip angle of coal seam; *c*_*0*_ and *φ0* are the cohesion and internal friction angle of coal pillar/filling column and roof and floor respectively.

In order to ensure the stability of coal pillars, it is necessary to meet *Fp* > *Fs*, that is:

16 $$b \ge \frac{{6r_{p} \sigma_{p} + 6\gamma aH - 5\gamma a^{2} }}{{6\left( {\sigma_{p} - \gamma H} \right)}}.$$

Filling width b is generally equal to strip mining width a. While designing the width of strip mining, the stability of this width strip coal pillar/filling belt is checked by Formula (12). When it ≤ a, you can set b = a; when b > a, it is necessary to reduce the width of strip mining a, and then calculate the width of strip coal pillar/filling belt by formula (16) until b ≤ a is satisfied, and set b = a.

Through theoretical calculation, the limit width a of strip mining is 10 m. In order to make underground production safer, combined with the actual situation, the strip mining width a should be less than 10 m. According to the general situation of the project, the average buried depth of the 11,071 working face is 75 m, which is brought into Formula (13). The calculated final compressive strength of the filling body is not less than 3.7 MPa. In order to maintain the long-term stable bearing capacity of the filling body, the safety factor is 1.1–1.5, and the final compressive strength of the filling body is preliminarily designed in the range of 4.07–5.55 MPa.

According to the previous theoretical analysis, the width of filling zone b is equal to the width of strip mining a, and a = b < 10 m. Therefore, the filling design is shown in Table 1.

**Table 1:** Design of filling simulation scheme.

Filling scheme	All falling down	9 m apart filling 9 m	6 m apart filling 6 m	3 m apart filling 3 m	Complete filling
Filling ratio	0	46	50	50	100

## Filling material test

### Filling material composition test

**Figure 5:**
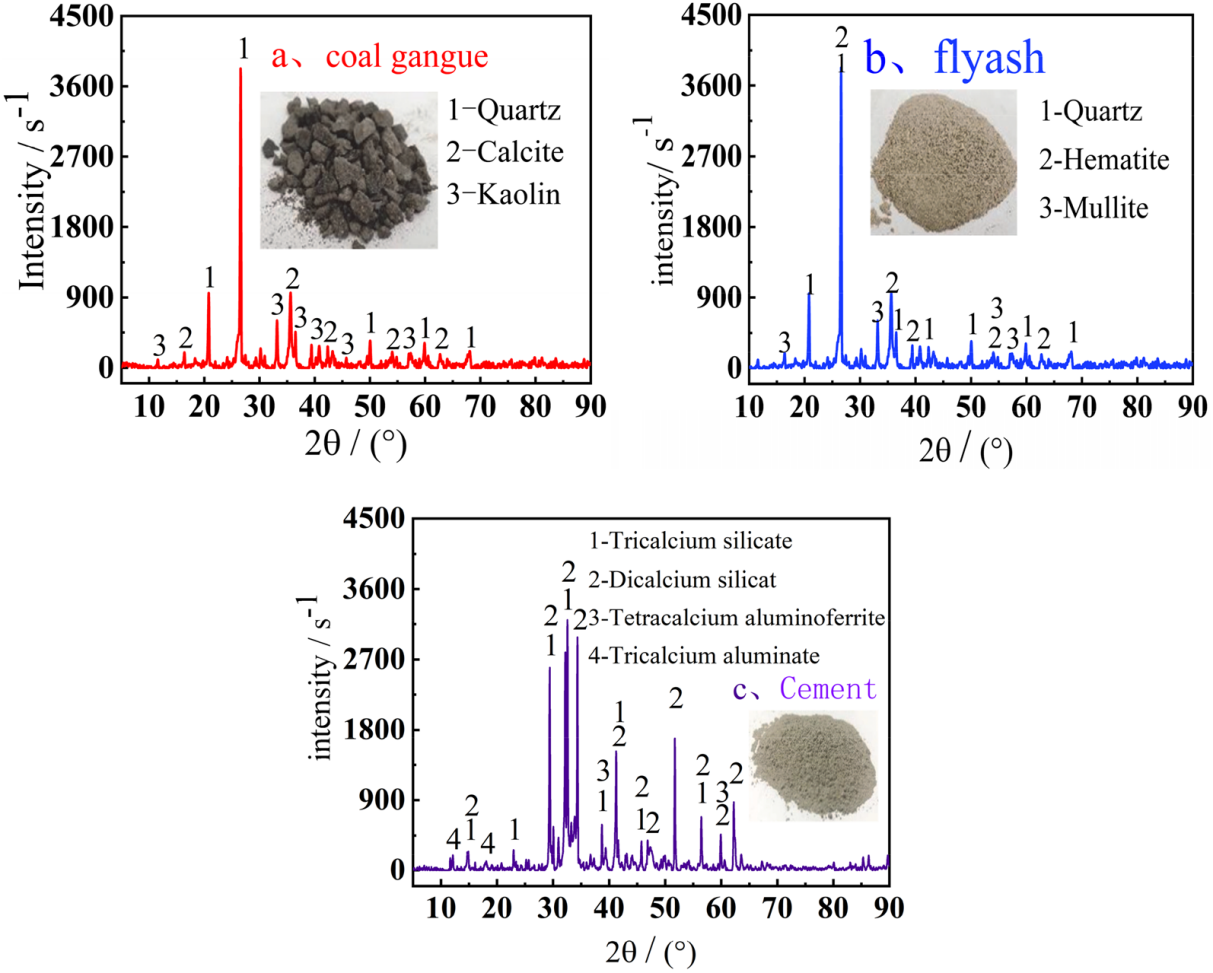
XRD analysis of mineral components.

Carbon gangue In mining regions, Portland cement, fly ash, and coal gangue make up the majority of the cemented infill materials. A coal gangue pile, a power plant, and a building materials industry in Guizhou are all used to produce cement, fly ash, and fly ash. Figure 5 displays the results of an XRD study of the mineral composition of raw materials.

Coal gangue contains a significant amount of quartz, which can be employed as filling aggregate to guarantee the strength of the filling body. Dicalcium silicate, tricalcium silicate, tricalcium aluminate, etc. are all widely present in cement. The strength of the cemented backfill will be significantly increased by the hydration of these minerals. Certain active components included in fly ash encourage hydration reaction and increase the strength of the filling body.

### Mechanical strength test

Fly ash and cement are combined to create a cementing solution that uses less cement while still meeting filling strength requirements in order to lower the cost of filling. It is found through a research of the literature and an initial pre-experiment that a coal gangue aggregate mass ratio of 45% is ideal. The impact of fly ash mixing amount, cementing material mixing amount, and slurry concentration on the strength of filling material is also investigated by the author. The influencing factors A, B, and C are presumptively the mass concentration of filling slurry, the quantity of fly ash, and the quantity of cementing material. The range of the filling slurry's mass concentration is 78%, 80%, and 82%, and the fly ash to cementing material ratio is 1.5 to 3.5:0.5 to 1.5. Table 2 depicts the ratio design.

**Table 2:** Table of standard specimen proportioning scheme under different factors.

Specimen number		Material ratio		Mass concentration /%	Expansion degree/mm
		Flyash	Binding material		
A	A1	2.5	1	78	255
	A2	2.5	1	80	231
	A3	2.5	1	82	209
B	B1	1.5	1	80	247
	B2	2.5	1	80	231
	B3	3.5	1	80	225
C	C1	2.5	0.5	80	243
	C2	2.5	1	80	231
	C3	2.5	1.5	80	210

According to the ratio, gangue, fly ash, and cement were weighed, then thoroughly agitated for 20 s without adding water. Water was then added, stirred for 60 s, and then slowly poured into a triple cubic mold with dimensions of 70.7 70.7 70.7 mm. The appliance's surface material was cleaned, and it was then placed in a curing box with consistent humidity and temperature. 20 °C was maintained, there was a minimum relative humidity of 95%, and the curing times were 3 days, 7 days, and 28 days, respectively. When the filling had reached the curing age, a press was used to gauge its strength.

**Figure 6:**
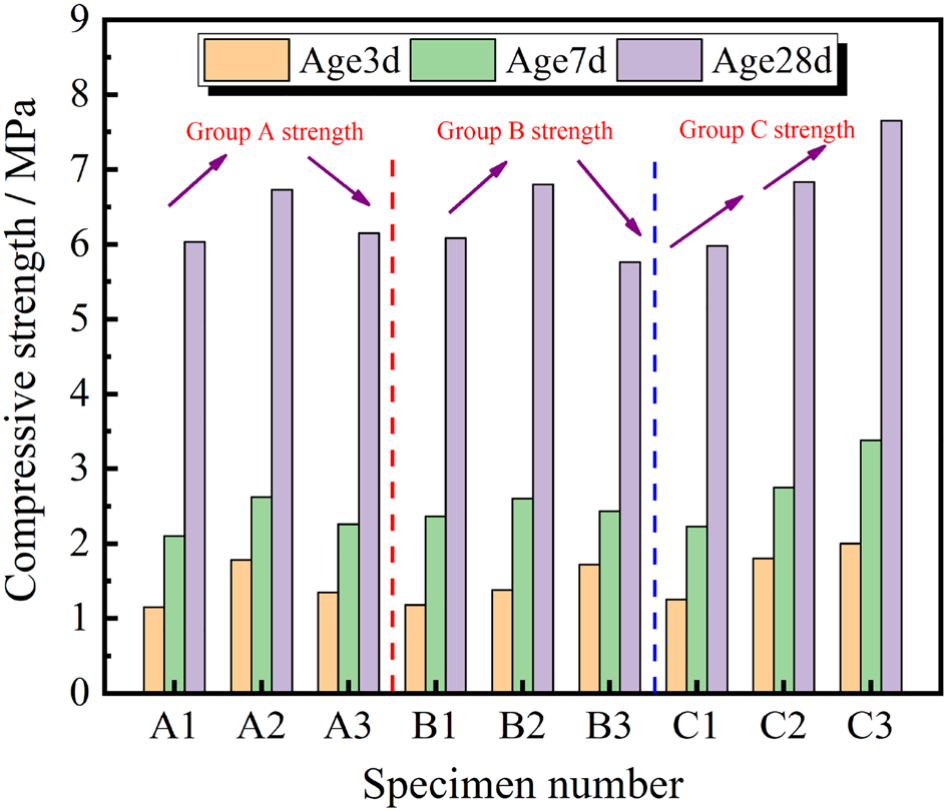
Relationship between uniaxial compression strength and influencing variables A, B, C.

When the mass concentration of the filling slurry in group A is less than 80%, it is evident from the expansion test results in Table 2 and the uniaxial compression test results in Fig. 6 that the uniaxial compressive strength increases with the concentration. The uniaxial compressive strength will be somewhat reduced when it exceeds 80%. The internal viscosity of the filling body will be impacted by the excessive concentration, and the encapsulation of water molecules by fine particles like cement and fly ash in the slurry will worsen. This will have a negative impact on the hydration reaction and lower the uniaxial compressive strength. The final strength (28-day strength) can therefore reach 6.75 MPa with a mass concentration of 80% when all other circumstances are certain; Internal filling body viscosity in group B shifts from dilution to viscosity as fly ash content rises. The compressive strength of the filler body increases as the fly ash content rises from 1.5 to 2.5 when the proportion of fly ash content is changed. The strength of the filler body varies little initially when the fly ash percentage is increased from 2.5 to 3.5%. The high internal viscosity causes a loss in compressive strength at a later stage, and the ideal fly ash content is about 2.5; With more cement present in group C, the strength is likewise rising, demonstrating cement's dominant position. In addition, the alkaline environment activates the gangue fly ash's active ingredients, enhances the hydration response, and significantly strengthens the filling body. The 10% cement percentage is the optimal option when taking into account cost and assuming that the on-site filling strength would be met.

### Hydration mechanism

**Figure 7:**
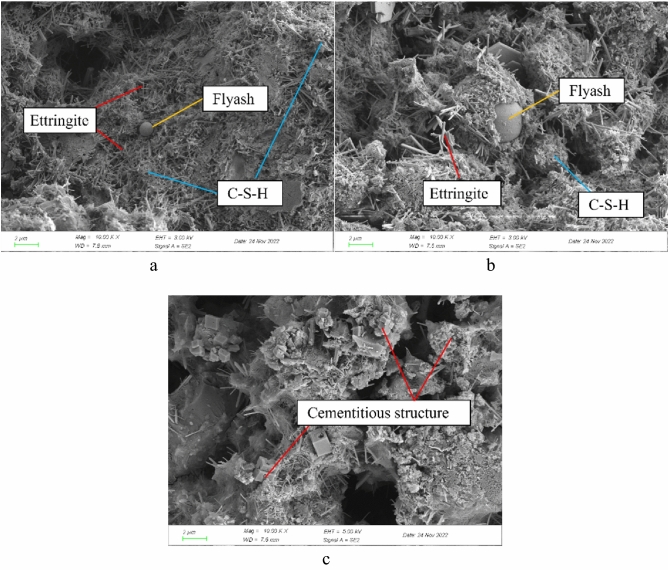
Electron microscope scanning SEM images of filling paste. (**a**) Age 3 days, (**b**) age 7 days, (**c**) age 28 days.

The kind, spatial distribution, quantity, relative size, and other properties of the hydration products have an impact on the filling body's strength, which changes as its internal properties change. The following elements^18,19^ are the primary components of the internal hydration process: in order to explore the microstructure changes of filling specimens with ages of 3 days, 7 days, and 28 days, the surface was observed under an electron microscope. The results showed that: (1) fly ash activity is stimulated by the hydration products of cement, and new substances are formed after the reaction; (2) insufficiently reactive semi-substances; (3) not involved in the reaction of primary material and its pores and microcracks. Figure 7 displays the SEM findings.

The SEM photos of the filling paste at various curing ages in Fig. 7 show that there are numerous needle-like structures on the surface of the specimen when the curing period is 3 days, but there is no visible interweaving phenomenon. Ettringite makes up the structure, and there are also some flocculent and sheet-like morphological structures. The ingredients are, respectively, C–S–H gel and Ca(OH)2. The specimen's gap is where the black hole is located, and fly ash fills the central sphere. When the curing time exceeds 7 days, the internal needle-like products get shorter and finer, the spherical fly ash gets wrapped up more securely, the amount of flake-like Ca(OH)2 rises significantly, and the C–S–H gel and needle-like ettringite get mixed up. The entire specimen exhibits denser, C–S–H gel and needle-like ettringite grow and form a full gel by the time the curing period reaches 28 days^20,21^. As a result, as people age, their needs for hydration shift, and as they age more, they become entangled and form a dense structure that increases the whole body's strength.

## Numerical simulation

### Establishment of numerical model

**Figure 8:**
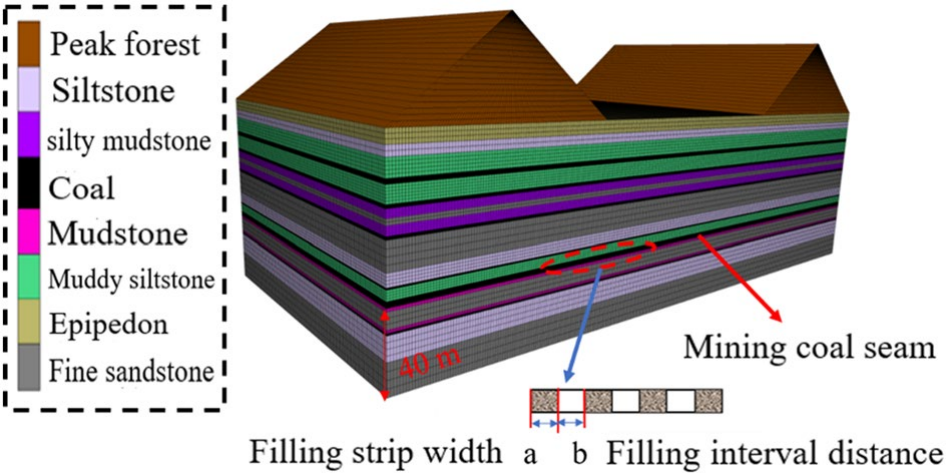
Numerical model.

The FLAC3D numerical simulation software is used to streamline the modeling in accordance with the geological conditions and rock parameters of the mining area, as indicated in Table 3. The Mohr–Coulomb strength criterion is used in this model's solution of the constitutive relationship. The model has a striking length of 400 m and a dip length of 200 m. The height *h*_*1*_ of the non-surface undulation is 115 m. At the same time, the peak height *h*_*2*_ on both sides of the strike is 50 m, and the central portion of the strike is a 150 m plain region b (also the valley bottom area). The 11,071 working face is 40 m from the bottom of the model and measures 300 m in length in x and 100 m in y directions. 50 m protecting coal pillars are positioned on either side of the x and y directions to help eliminate the impact of boundary conditions. The materials for the model are assigned, and the boundary is limited, based on the mechanical characteristics of the coal, rock mass, and filler body. Each of the five mining plans listed in Table 1 is simulated. To track the displacement and stress state of the roof, measuring sites are evenly spaced along each monitoring line at heights of 0 m, 6 m, 12 m, 18 m, and 24 m above the coal seam. Three monitoring lines are simultaneously set up at the bottom, top, and middle of the filling body to track its stress level. In Fig. 8, the numerical model is displayed.

**Table 3:** Physical and mechanical parameters of coal strata.

Rock formation	Density/(kg m^−3^)	Shear/GPa	Bulk /GPa	Cohesion/MPa	Tensile /MPa	Friction/°
Silty mudstone	2417	5.7	10.76	1.18	1.46	35
Siltstone	2460	8.13	10.83	2.75	1.84	38
Fine sandstone	2600	11.2	15.28	3.1	3.48	42
Coal	1460	0.93	2.12	0.5	0.35	24
Muddy siltstone	2400	3.8	5	4	2.5	35
Mudstone	2250	2.27	4.39	2.9	1.8	25.2
Epipedon	1960	0.098	0.28	0.85	0.35	25
Obturator	2560	1.05	1.05	3.2	1.05	27

### Displacement monitoring results

**Figure 9:**
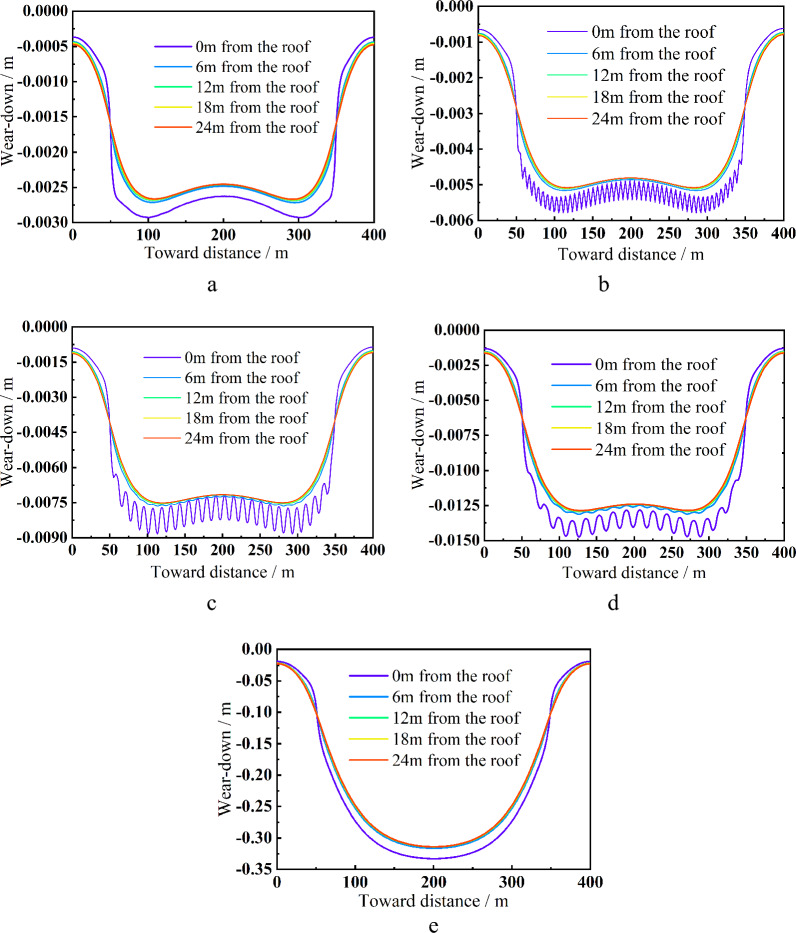
Roof settlement displacement curve of each scheme. (**a**) All filling down, (**b**) 9 m apart filling 9 m, (**c**) 6 m apart filling 6 m, (**d**) 3 m apart filling 3 m, (**e**) complete filling.

Figure 9 shows that when the stope is entirely filled, the immediate roof's maximum subsidence does not exceed 3 mm, and the overlying strata's sinking trend from 6 to 24 m away from the roof exhibits almost the same trends. Between 2.5 mm and 3 mm is the median range of subsidence. The overlying strata's overall subsidence curve has a "W" form, and the mountain is where there is the most subsidence. When the "filling 3 m with 3 m interval" plan is used, the immediate roof's maximum subsidence is around 6 mm, and its settlement curve has a "wave" appearance. The maximum sinking area gradually moves to the stope's middle at this point, whereas the overlying strata's subsidence trend from 6 to 24 m is essentially the same and the subsidence range in the stope's center is between 4.8 and 5.1 m. When the "6 m interval filling 6 m" method is employed, the immediate roof's maximum subsidence is about 9 mm, and the "wave" settlement tendency of the immediate roof is more visible, suggesting that the immediate roof has sustained more substantial overall tensile and shear damage. The overlaying strata's settlement curve gradually transforms from a "W" shape to a "basin" shape, with a center settlement range of between 7.1 mm and 7.5 mm; When the "interval 9 m filling 9 m" is used, the immediate roof's maximum subsidence is about 15 mm, and the "wave" shape settlement curve becomes sparse, indicating that the immediate roof's overall tensile and shear damage is further increased, and the distance from the roof is increased from 6 to 24 m. The centre of the overlaying strata in the stope settle between 12.1 mm and 12.5 mm, and the settlement curve's form remains rather constant. The middle stope experiences the largest subsidence, which is around 340 mm at 0% filling rate, and the settlement curve takes on a "U" shape. In conclusion, a sensible filling mining strategy can successfully manage the stability of the roof. The maximum subsidence of the immediate roof drops from 340 to 3 mm from a filling rate of 0–100%, and the decline is noticeable. The settlement curve shifts from a "U" to a "basin" and then to a "W" form. The direct roof settling curve takes on a "wave" structure when local filler mining is done, and the fluctuation amplitude steadily decreases.

### Stress monitoring results

**Figure 10:**
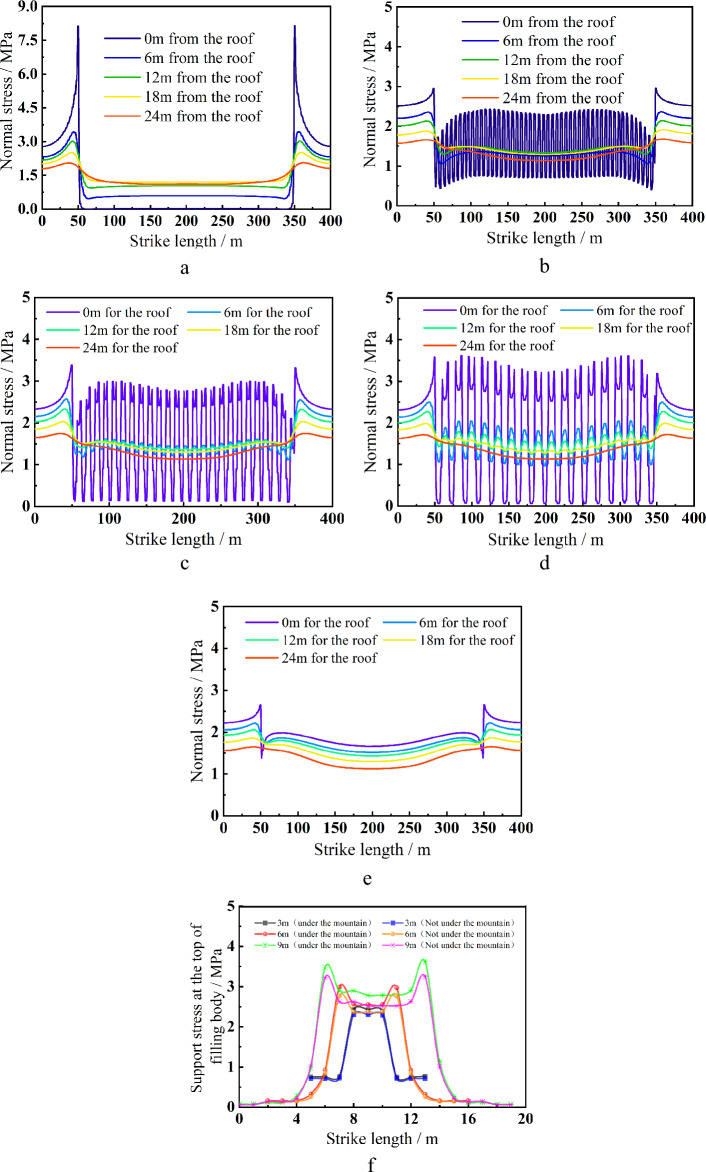
Vertical stress variation curve of roof and local filling body top in each scheme. (**a**) All filling down, (**b**) 9 m apart filling 9 m, (**c**) 6 m apart filling 6 m, (**d**) 3 m apart filling 3 m, (**e**) complete filling, (**f**) vertical stress distribution at the top of filling body.

Figure 10 illustrates how the stope's coal wall experiences stress concentration on both sides when it is completely filled. At this point, the overlying strata are moving toward their original rock condition because to the 2.7 MPa maximum vertical stress. The pressure relief condition at the overlying strata conforms to the stress distribution of the overlying strata in the "three zones" theory when the stope is not filled, and the vertical stress peak on both sides of the coal wall reaches roughly 8 MPa. When local filling mining is used, a 'cycle' can be seen in the stress curve of the direct roof in the middle of the stope. With the reduction in filling spacing, the peak stress on both sides of the stope exhibits a modest decreasing trend. Without zero bearing load, the vertical stress progressively transforms from a "saddle" shape to a "inverted U" shape at the top of the filling body. The stress valley value in the middle of the stope is close to 0 MPa when the filling spacing is 9 m, demonstrating that the immediate roof in the vacant roof area effectively loses its bearing capacity. Greater than the peak stress of 3.2 MPa in the middle of the stope, the filling body's peak stress of 3.65 MPa is under the valley region. The immediate roof in the empty roof region still has a weak bearing capacity when the filling spacing is 6 m, but the cooperative filling body can sustain the immediate roof by acting as a filling body when the stress valley value in the middle of the stope is close to 0.3 MPa. 2.98 MPa, which is higher than the 22.7 MPa peak stress in the middle of the stope, is the peak stress of the filling body beneath the valley area. The stress fluctuation steadily reduces as the measuring line's height rises; it only changes below the roof's 18-m measurement line. The stress valley value in the middle of the stope is close to 0.5–0.7 MPa when the filling spacing is 3 m, which demonstrates that the immediate roof in the vacant roof area has a certain bearing capability. 2.55 MPa is higher than the peak stress in the center of the stope for the filling body beneath the valley area. 2.42 MPa, the change in stress fluctuation steadily decreases as the height of the survey line rises, and the stress change is only visibly noticeable below the 6 m survey line from the roof.

### Plastic-zone analysis

**Figure 11:**
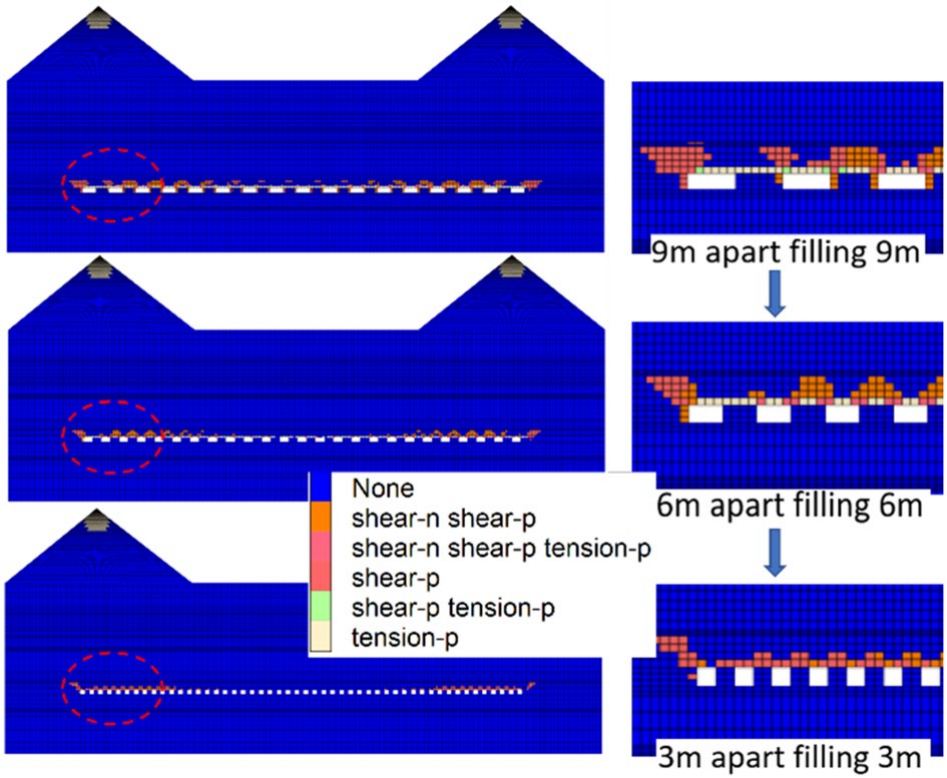
Plasticity distribution map of local filling scheme.

The overall plastic zone of the stope is the largest when the filling interval is 9 m, and the tensile and shear damage regions of the surrounding rock of the branch roadway are also the largest, as shown in Fig. 11. At this point, it is challenging to guarantee the long-term stability of the filling support body since plastic zones measuring roughly 1 m on either side of the edge of the filling body are visible. While the plastic zone under the mountain area is larger than that in the middle area of the stope, the range of the entire plastic zone of the stope is obviously reduced, and the immediate roof's and the filling body's combined bearing essentially guarantees the stability of the surrounding rock. The plastic zone is further diminished when the spacing is lowered to 3 m, and only a minor amount of tensile and shear failure exists on both sides of the filling body beneath the mountain, which has a positive control impact on the strata above. As a result, the surface load effect is clear and the stability of the "filling strip-direct roof" composite structure is compromised by the wider filling spacing's tendency to increase the tensile and shear damage range of the vacant roof area and both sides of the top.

## The engineering test

### The filling process

**Figure 12:**
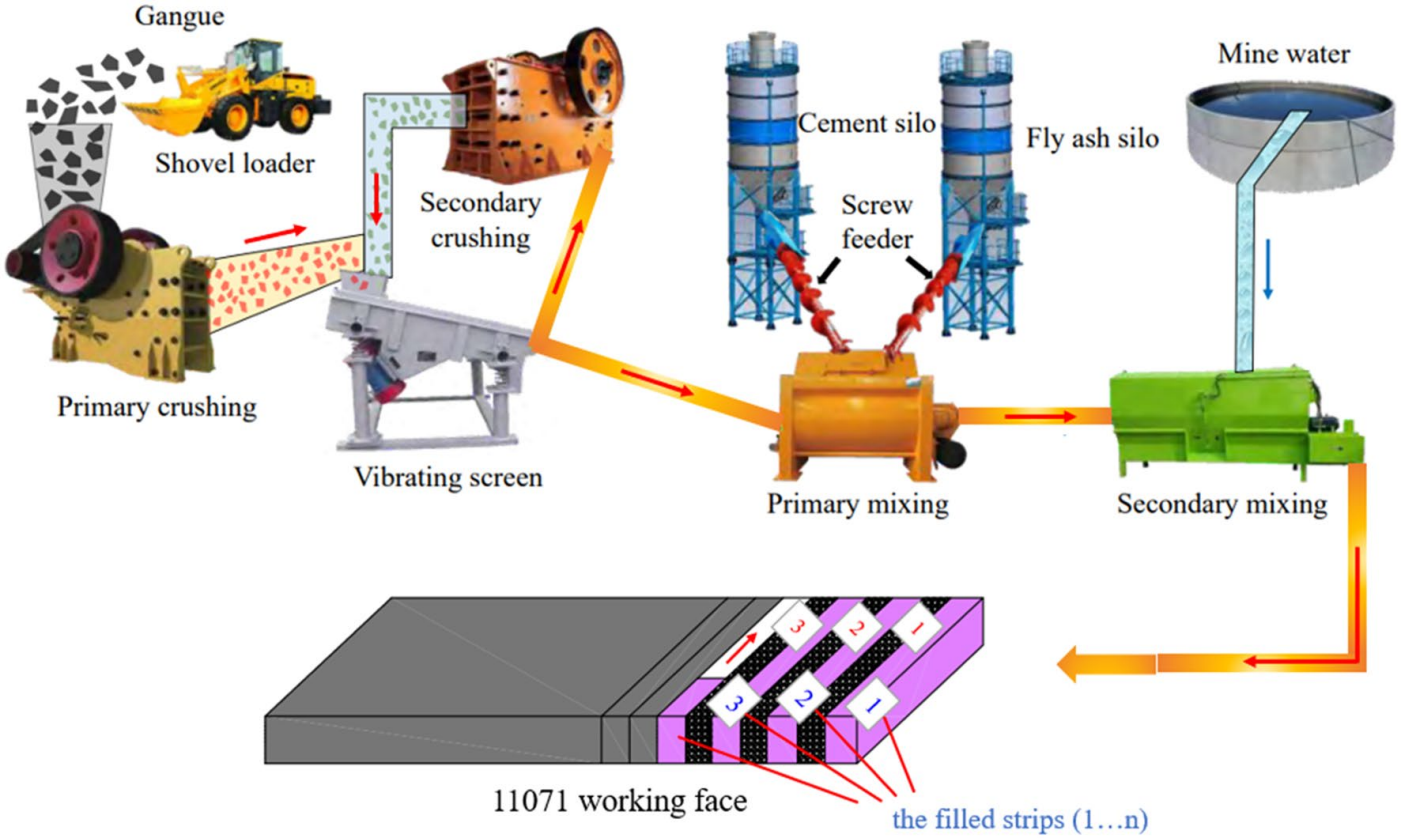
Filling process flow chart^22^.

To meet the requirements for particle size, gangue from the deputy well was selected, transported to the crushing plant using dump trucks, fed through the hopper into the crusher in two stages, with the roller crusher and high-fine crusher for the first and second stages, respectively. Fly ash, cement, and other filling materials were then loaded into their residence using forklift trucks. The slurry production system consists of two levels of mixers: the counter-rotating mixer for finely mixing slurry and the vane mixer for coarsely combining gangue, fly ash, cement, and other dry ingredients. Water for the second mixing should be added to the mixer after measurement using the pump to draw water from a 30 m^3^ reservoir. The necessary filling material is discharged into the conveying pump and pushed into the conveying pipeline to fill the 11,071 spacing strips on the working face after being thoroughly mixed in the mixer. The filling process is shown in Fig. 12.

### The engineering monitoring

**Figure 13:**
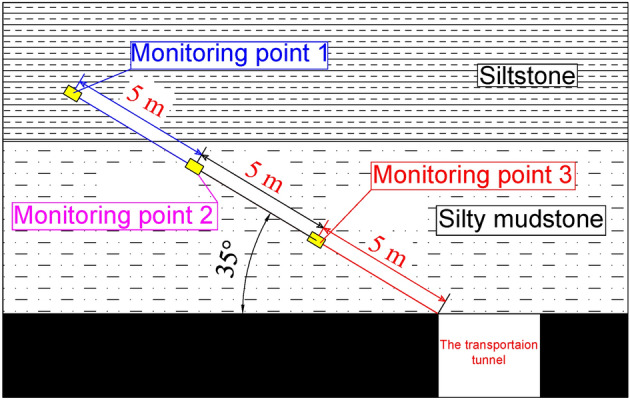
Monitoring station borehole layout profile.

The local filling mining scheme of 'filling 3 m interval 3 m' is used in the field based on theoretical and numerical simulation studies. Six monitoring stations are set up underground in the middle of the transportation lane to further test the filling effect. The distances from the open-off cut are 30 m, 60 m, 90 m, 120 m, 150 m, and 180 m, and the movement of the above strata is monitored during the mining process. The monitoring boreholes are positioned at a 35-degree elevation angle, with three measuring stations 5 m, 10 m, and 15 m from the orifice, Fig. 13 depicts a profile of the monitoring station borehole layout. Table 4 displays the monitoring findings.

**Table 4:** Roof displacement monitoring statistics of different monitoring stations.

Measuring point	Displacement of different monitoring stations /mm					
	30	60	90	120	150	180
Measuring point 1	1.25	1.0	0.9	1.0	1.2	1.1
Measuring point 2	3.7	3.2	2.3	2.9	3.2	3.6
Measuring point 3	10.9	10.3	8.5	10.2	10.6	10.8

The monitoring reveals that the displacement of each monitoring station differs following the end of the filling mining face mining. The bigger the displacement of the measurement point, the closer it is to the stope, and the greater the displacement, the higher the position of the measuring line. The overall displacement of the measuring point is as follows: measuring point 3 > measuring point 2 > measuring point 1. The overall displacement of the station at 30 m from the open-off cut is greater, the greatest displacement is 10.9 mm, and then falls and then rises. This is due to the effect of surface load. The surface mountain area corresponds to the 30 m position from the open-off cut, which conforms to the numerical simulation findings. In general, the movement of the overlaying strata is not significant, which is consistent with engineering practice.

## Conclusion

1. A full negative pressure ventilation working face is built in accordance with the configuration of the wall mining production system, and mining is done in accordance with the sequence of "filling strip, 1-caving strip, 1-filling strip, 2-caving strip, 2-…-filling strip n." The width of the filling strip and the width of the strip mining are designed to be equal in order to maintain the steady bearing of the filling strip. The fracture step distance of the immediate roof is around 10 m, and the width of the infill strip and the interval distance should both be less than 10 m, according to the field investigation and theoretical analysis.
2. The internal viscosity of the filling slurry will increase in accordance with the indoor test when A, B, and C influencing factors increase, however going over a particular threshold will have an impact on the hydration response. The best fly ash contributes 25%, while the best cementing material contributes 10%; the ideal slurry concentration is 80%, and the final strength can reach 6.75 MPa. When the SEM diagram is added, it is clear that as the curing age increases, more needle-like and flocculent products are formed by the hydration process, intertwining and encasing fly ash and gangue particles to form a full cementitious structure. The entire specimen is solid and strong enough.
3. It can be seen by numerical simulation that the reasonable filling mining strategy can successfully manage the stability of the roof. The maximum subsidence of the immediate roof is decreased from 340 to 3 mm from a filling rate of 0–100%, and the decrease is noticeable. The settlement curve shifts from a "U" to a "basin" and then to a "W" form. The immediate roof's settlement curve has a "wave" appearance. The immediate roof's stress curve displays a steady "cycle" in the middle of the stope. With a reduction in filling spacing, the peak stress of the coal wall on both sides of the stope somewhat lowers. There is no zero bearing load and the vertical stress progressively transforms from a "saddle" shape to a "inverted U" shape at the top of the filling body. In addition to the plastic zone, it is evident that the filling spacing affects the tensile and shear damage range of the empty roof area and the top sides. This decreases the surface load effect and promotes the stability of the "filling strip-direct roof" composite structure.
4. According to on-site monitoring, the displacement increases with increasing distance between the measurement point and the stope while decreases with increasing measuring line height. Overall, the measuring point displacement is as follows: measuring point 3 > measuring point 2 > measuring point 1.The highest displacement is just 10.9 mm, which is consistent with the numerical modeling, however the displacement beneath the mountain area is excessively great while the overall displacement of the strata above is negligible.

## Data Availability

The datasets generated and analysed during the current study are not publicly available due our whole study is still in its early stages, and future research will build on the information we have so far, but are available from the corresponding author on reasonable request.
